# Response to letter regarding comment on “worldwide prevalence of haemorrhoids: a systematic review and meta-analysis”

**DOI:** 10.1080/07853890.2026.2641284

**Published:** 2026-03-07

**Authors:** Pouyan Ebrahimi

**Affiliations:** ^a^ School of Medicine, Babol University of Medical Sciences, Babol, Iran; ^b^ Cellular and Molecular Biology Research Center, Health Research Institute, Babol University of Medical Sciences, Babol, Iran; ^c^ Cancer Research Center, Health Research Institute, Babol University of Medical Sciences, Babol, Iran

Dear Editor

We sincerely thank Dr. Wang for his thoughtful and constructive comments on our systematic review and meta-analysis [1]. We appreciate the opportunity to clarify several methodological aspects of our work.

First, regarding the substantial heterogeneity observed across prevalence estimates (I^2^ = 100%), we fully agree that high heterogeneity is inherent to large-scale prevalence meta-analyses [2], particularly when synthesizing data from diverse populations, healthcare systems, and diagnostic approaches. Given the broad geographic scope of our study, encompassing 150 studies across 45 countries, substantial between-study variability was expected. We conducted extensive subgroup and multivariable meta-regression analyses to explore potential sources of heterogeneity using all available study-level data. As reported, the included covariates explained 16.86% of the between-study variance, indicating that additional unmeasured factors likely contribute to the residual heterogeneity. Importantly, we explicitly acknowledged this limitation in both the discussion and conclusion sections and emphasized that pooled estimates should be interpreted cautiously. This is particularly relevant for country-level estimates based on single studies, which we noted as indicative of the need for further primary research rather than definitive national prevalence figures.

Second, concerning the presentation of risk factors, we clearly stated throughout the manuscript, including in abstract, result (Table 4), discussion, and limitations, that these associations were derived from unadjusted estimates. These analyses were not the primary objective of our study but were synthesized from a limited subset of 16 studies. We consistently framed these findings as exploratory and hypothesis-generating, intended to guide future prospective and mechanistic research. We agree that confounding may influence these associations, consistent with established methodological guidance [3,4], and explicitly cautioned readers against causal interpretation. Factors such as family history and pregnancy, which demonstrated significant associations, warrant further evaluation in well-designed longitudinal studies.

Third, regarding the temporal trend and the possibility of diagnostic intensification, it is important to clarify that the reported annual increase in prevalence emerged from the multivariable meta-regression model, not from univariable analysis. In the univariable model, publication year was not significantly associated with prevalence. However, after adjustment for relevant covariates, including diagnostic method (with non-invasive methods treated as the reference category), the association became significant, suggesting that confounding was addressed in the multivariable framework. In response to this comment, we conducted an additional sensitivity analysis stratified by diagnostic method. The pooled prevalence for non-invasive methods ranged from 21.70% to 23.53%, and for invasive methods from 27.20% to 28.41% (all leave-one-out analyses, *p* < 0.001). Although invasive methods yielded numerically higher estimates, no statistically significant difference was observed between invasive and non-invasive approaches overall (*p* = 0.145). These findings suggest that improved diagnostic capacity alone is unlikely to fully explain the observed temporal pattern. As discussed in the original article, broader lifestyle transitions, including population aging, which has been associated with higher prevalence rates in epidemiological studies [5], as well as increased sedentary behavior and dietary westernization, may also contribute.

We are grateful to Dr. Wang for his careful evaluation and valuable observations, which have strengthened the interpretation of our findings and contributed meaningfully to this ongoing scientific discussion.

## Data Availability

Data sharing is not applicable to this article as no data were created or analyzed in this research.
